# Major Perioperative Cardiac Risk Assessment: A Review for Cardio-Oncologists and Perioperative Physicians

**DOI:** 10.3390/clinpract14030071

**Published:** 2024-05-17

**Authors:** Emily P. Johnson, Robert Monsour, Osama Hafez, Rohini Kotha, Robert S. Ackerman

**Affiliations:** 1 Herbert Wertheim College of Medicine, Florida International University, Miami, FL 33199, USA; ejohn138@med.fiu.edu; 2 Morsani College of Medicine, University of South Florida, Tampa, FL 33602, USA; rcmonsour@usf.edu; 3 Department of Anesthesiology, Moffitt Cancer Center and Research Institute, 12902 USF Magnolia Drive, Tampa, FL 33612, USA; rohini.kotha@moffitt.org (R.K.); robert.ackerman@moffitt.org (R.S.A.)

**Keywords:** perioperative medicine, cardio-oncology, anesthesiology, cardiology, postoperative outcomes

## Abstract

The Revised Cardiac Risk Index (RCRI) and the American College of Surgeons (ACS) National Surgical Quality Improvement Program (NSQIP) preoperative risk assessment tools are the most widely used methods for quantifying the risk of major negative perioperative cardiac outcomes that a patient may face during and after noncardiac surgery. However, these tools were created to include as wide a range of surgical factors as possible; thus, some predictive accuracy is sacrificed when it comes to certain surgical subpopulations. In this review, we explore the various surgical oncology patient populations for whom these assessment tools can be reliably applied and for whom they demonstrate poor reliability.

## 1. Introduction

When undergoing noncardiac surgery, patients are at an increased risk of major adverse cardiovascular events (MACEs), particularly those undergoing high-risk operations, ischemic heart disease, congestive heart failure, cerebrovascular disease, and insulin therapy, or displaying creatinine levels >2.0 mg/dL [1]. The RCRI and ACS NSQIP assessment tools attempt to calculate the cardiovascular risk that a particular surgery poses based on both patient and surgical characteristics. Specifically, the RCRI utilizes six patient and surgical factors to determine the 30-day postoperative probability of death, myocardial infarction (MI), or cardiac arrest in patients undergoing nonurgent major noncardiac surgery (Table 1) [1].

The ACS NSQIP similarly takes into account both patient and surgical factors, including twenty-one factors in total (Table 2). 

While the inputs of each of these factors have all been found to be independently predictive of postoperative outcomes, often generating reliable postoperative risk assessments when integrated, these calculators might not be very applicable to cancer patients. This is because patients undergoing oncologic surgery often possess unique risk factors, such as exposure to neoadjuvant chemotherapy with cardiotoxic effects or increased frailty due to cachexia. These issues increase patients’ risks beyond what these calculators might estimate [2,3]. In addition, patients with cancer were not well represented in previous perioperative risk assessment trials [1,3]. Thus, it is important to understand which cancer patient subpopulations can continue to rely on the risk assessments of these two calculators and which would benefit from additional considerations when weighing risks and benefits before proceeding with a surgical treatment plan.

In this paper, utilizing the RCRI and ACS NSQIP, we address the populations of surgical oncology patients whose perioperative cardiac risk can be accurately accounted for. Utilizing the National Library of Medicine’s online database, we searched for peer-reviewed articles that utilized search words including “surgical oncology”, “neoadjuvant therapy”, “Revised Cardiac Risk Index”, and/or the “National Surgical Quality Improvement Project”. Studies were excluded if cardiac oncology or cardiac surgery was the main operative focus. 

### 1.1. Revised Cardiac Risk Index (RCRI)

Concerns about the RCRI’s application to oncologic surgery patients have been raised since its inception, as the derivation study did not consider an indication for oncology-related surgery nor the diagnosis of cancer. Interest in this topic was further piqued because an international prospective cohort study found that the RCRI underestimated the 30-day postoperative MACE rates by a factor of six when the surgery was conducted at a dedicated cancer center [4]. As the RCRI considers the body compartment that is undergoing surgery, and as oncologic surgeries are usually performed for cancers that consistently arise in single body compartments, we approached our evaluation of this calculator on the basis of body compartment.

For abdominal surgery, one study on patients undergoing elective gastrectomy for gastric cancer found that in the 30-day postoperative period, the RCRI score was independently predictive of MACEs, with most events occurring on postoperative day (POD) 2 or earlier [5]. In addition, the RCRI was independently predictive of in-hospital mortality and pneumonia during this time, but unrelated to other local surgical complications like anastomotic leaks. Amongst the other measured factors, only tumor stage was found to be an independent predictor of MACEs. Although total gastrectomies demonstrated increased MACEs relative to distal gastrectomies, the differences were not statistically significant (*p* = 0.055). 

For colorectal cancer, a multicenter retrospective cohort study found that the RCRI score was linearly related to 90-day postoperative mortality; cancer stage also demonstrated a similar proportional relationship with mortality [6]. In terms of operative factors, the extent of resection was not found to significantly affect immediate postoperative mortality, but a minimally invasive approach was associated with a lower 90-day mortality compared to the use of an open approach [6]. Of note, neoadjuvant therapies, including both chemotherapy and chemoradiation, were not associated with an increase in postoperative mortality. However, it must be noted that this treatment modality was mostly used in patients with a score of 1 [6].

Patients who underwent elective surgery for gastrointestinal, hepatobiliary, or pancreatic malignancies found that an RCRI score ≥3 was independently associated with increased 30- and 90-day postoperative mortality rates [7]. More specifically, in patients with RCRI scores of less than three, the 30-day postoperative mortality rate was 2%, whereas patients with RCRI ≥ 3 had an 8.5% mortality rate; subgroup analysis showed that patients undergoing upper gastrointestinal surgery also suffered from a significantly higher 30-day mortality rate than those undergoing lower gastrointestinal surgery, and a surgery duration of greater than 130 min was independently associated with an increased total complication rate. 

When reviewing these external validation studies of the RCRI for intra-abdominal oncologic surgery, it is apparent that this calculator is reliable in predicting 30-day MACEs. The RCRI’s predictive range might even be extended to other outcomes, such as pneumonia [5,7]. Even greater predictive utility might be achieved by incorporating other factors such as tumor stage, particularly that of stage III or greater, into models. This is important given its independent association with increased MACE rates—possibly due to its frailty and its potentiation of MACEs [5,6]. Additionally, while the extent of resection was not found to be significantly associated with increased MACE or mortality rates in any of these reviewed studies, an open operative approach [6] and a surgery length of greater than 130 min [7] were associated with increased mortality and complications, respectively. Thus, while intraperitoneal surgery is accounted for by the RCRI as an elevated-risk surgery, including open surgery or extended expected operation time in the assessment might improve the accuracy of postoperative predictions. 

For intrathoracic malignancies, the RCRI accounts for the additional risk associated with operating in this body compartment. However, original RCRI studies included relatively few thoracic surgery patients, and thus there were already concerns about their applicability to this patient population at their inception [1]. One study found that in patients who underwent oncologic lung surgery, only three of the traditional six factors were significantly associated with 30-day postoperative MACEs: ischemic heart disease, creatinine > 2 mg/dL, and cerebrovascular disease [8]. Undergoing a pneumonectomy, as opposed to a lobectomy, was itself an independent predictor of MACEs. Additionally, because all six RCRI factors were weighted equally and all patients in this study underwent an elevated-risk surgery, the area under the receiver operating curve (AUC) of this calculator was reduced due to the reduced number of possible risk strata. A similar retrospective cohort study of patients undergoing elective lung cancer resection found that the RCRI was a poor predictor of MACEs and showed a similarly reduced AUC due to risk strata loss [9]. 

For these reasons, Brunelli et al. created the Thoracic Revised Cardiac Risk Index (ThRCRI), which considers serum creatinine, ischemic heart disease, cerebrovascular disease, and pneumonectomy versus lobectomy, with different weighting for each factor [8]. This tool was found to provide superior discrimination compared to the traditional RCRI, especially amongst the highest-risk strata of patients (those with an RCRI ≥ 3) who had an observed cardiac complication rate three times higher than what was predicted by the RCRI. In subsequent validation studies into patients undergoing the resection of non-small-cell lung cancer, the ThRCRI class was associated with an increased incidence of cardiopulmonary morbidity and MACEs within 30 days of the operation [10,11]. Thus, it appears that the RCRI might benefit from incorporating the ThRCRI’s additional factors. However, it must be noted that Wotton et al. found that the ThRCRI still demonstrated difficulty in assessing the risk of 30-day mortality and MACEs [9]. As such, it appears that both the RCRI and ThRCRI require further study to determine if considering other factors might be more beneficial for cardiac risk assessments before lung cancer resection. 

Regarding retroperitoneal surgery, one study investigated the generalizability of the RCRI to patients with renal cell carcinoma undergoing partial nephrectomy [12]. The RCRI was found to perform poorly, with only the combination of ischemic heart disease and heart failure under the umbrella category “heart disease” being predictive in analysis. However, a serum creatinine level of >1.5 mg/dL, a value that is lower than the cutoff of 2 mg/dL in the RCRI, was most strongly associated with 30-day postoperative MACEs and mortality. This association between serum creatinine and outcomes demonstrates a unique correlation between renal function and the progression of renal cell carcinoma—a relationship that has been previously shown and which may function as a proxy of overall disease progression outside of the tumor stage [13]. 

Furthermore, this study found that four other factors in addition to “heart disease” and serum creatinine >2 mg/dL were predictive of outcomes in the univariate analysis: age ≥ 75 years, American Society of Anesthesiologists Physical Status score >2, anemia (hematocrit < 36% for females and <41% for males), and an open surgical approach. By equally weighing these six factors in a calculator, the authors found that this method demonstrated a superior discriminatory performance compared to the RCRI. Once again, it appears that unique disease conditions and the operative approach limit the application of a general-use calculator for risk assessment. 

Finally, the RCRI was applied in cardiac risk assessments for patients undergoing the resection of head and neck squamous cell carcinoma [14]. In these patients, the RCRI demonstrated a negative prediction trend for 30-day MACEs and mortality. Furthermore, in univariate analysis, four of the six RCRI factors were not associated with postoperative outcomes; of note, no subjects were found to have a serum creatinine content >2.0, and head and neck surgery was not considered to pose an “elevated risk”. Only operating room time was found to predict postoperative MACEs and mortality in both univariate and multivariate analyses. Thus, another example of operative factors—specifically, operating room time—has a relation to patient outcomes in a more consistent manner than RCRI scores. In specialized operations such as those that are required for the treatment of head and neck squamous cell carcinoma, where risk is not captured by calculators such as the RCRI, using more specialized cardiac risk assessment tools will likely be more beneficial than a general risk prediction framework. 

### 1.2. National Surgical Quality Improvement Project (NSQIP)

The ACS NSQIP was designed to enable surgeons to the estimate individualized risks of all surgical operations, utilizing an accessible format to counsel patients regarding elective surgeries and discuss patient-specific risks for emergent operations [3]. In a previous study, preoperative factors were selected a priori, with consideration of their predictive value, routine availability to surgeons prior to an operation, and clinical face validity [3]. Nearly 1.5 million cases, spanning nearly all surgical specialties, were evaluated by utilizing the ACS NSQIP database, and the ACS NSQIP was compared to previously published procedure-specific calculators [3]. The complication rates that were accounted for included serious complications (cardiac arrest, myocardial infarction, pneumonia, etc.), “any complication, pneumonia, cardiac complications, surgical site infection, urinary tract infection, venous thromboembolism, renal failure, colon ileus, colon anastomotic leak, readmission, return to the operating room, death, discharge to a nursing or rehab facility, and predicted length of hospital stay” [3]. The factors utilized to calculate risk using the ACS risk calculator (ACSRC) are listed in Table 2.

Of interest is the ACSRC’s inclusion of disseminated cancer as a risk factor, which the RCRI does not account for. Cancer and cardiotoxic neoadjuvant chemotherapy might complicate risk calculation for this specific group of patients. Thus, this component of the ACSRC offers the ability to predict risks more accurately for this oncologic patient population. Disseminated cancer was originally included in the ACS NSQIP’s colorectal risk calculator, the precursor to the universal ACSRC [15]. The inclusion of cancerous and non-cancerous colorectal procedures allowed for greater correlations to be established between the predictor variables. However, it is unclear how each predictor variable, including disseminated cancer, was selected [15]. The ACSRC guidelines for disseminated cancer risk inclusion are listed below:

“The patient has a primary cancer that has metastasized to a major organ and meets at least one of the following:

- A surgical procedure is the treatment for the patient’s metastatic cancer.
- The patient has elected to not receive treatment for the metastatic disease.
- The patient’s metastatic cancer has been deemed untreatable.
- The patient has disseminated cancer: acute lymphocytic leukemia, acute myelogenous leukemia, or stage IV lymphoma.”

In our review, we found seven studies that included trials that compared ACSRC preoperative risk assessment scores and 30-day postoperative outcomes across gastrointestinal, head and neck, thoracic, gynecological, and musculoskeletal oncologic specialties (summarized in Table 3).

The 30-day postoperative outcomes included rates of pneumonia, any complication (cardiac failure, other cardiac complications, etc.), cardiac complications, venous thromboembolism, and death. These were reported as Brier scores and C-statistics among other modalities of paper-specific statistical analysis. Positive predictive capability was determined based on a Brier score of less than 0.002 and a C-statistics score above 0.7. 

Of the seven studies assessed, three suggested that the ACSRC accurately predicts cardiac complication and death for total gastrectomies (Brier score = 0.019 and 0.017, respectively; C-statistic = 0.83), pulmonary resection (C-statistic = 0.821 and 0.753, respectively), and gynecologic laparotomies (Brier score = 0.011 and 0.008, respectively; C-statistic = 0.708 and 0.851, respectively) [17,20]. Another gastrointestinal study examining laparoscopic gastrectomies reported that ACSRC showed a moderate predictive capability for 30-day postoperative pneumonia and any complication (Brier score = 0.009 and 0.154, respectively; C-statistic = 0.65 and 0.57, respectively) [16]. However, cardiac complications were not predicted by the ACSRC in cases of other gynecologic surgeries (Brier score for any complication = 0.023) or proximal femur replacement in oncologic cases (C-statistic for any complication <0.576) [21,22]. Moderate predictive capabilities were also reported for head and neck oncologic operations [18]. 

These studies encompassed over 5000 oncologic clinical cases of varying Current Procedural Terminology (CPT) codes, covering gastrointestinal, head and neck, thoracic, gynecological, and musculoskeletal specialties. A positive predictive ability of the ACSRC for 30-day complications was found for gastrointestinal, thoracic, and gynecologic operations. In two studies, the ACSRC showed a strong or moderate predictive ability for gastrointestinal operations, indicating that it provides beneficial predictive preoperative risk assessment in oncologic gastrointestinal patients [16,17]. Although a predictive capability was found for gynecologic laparotomies, the ACSRC showed poor verification of cardiac complications with general gynecological surgeries, indicating the calculator’s heightened accuracy for noninvasive operations and its discrepancies across operations [20,21]. Lastly, the calculator was non-predictive of any complications for proximal femur replacements, suggesting potential limitations when applied to other oncologic orthopedic bone replacements [22]. 

Four of the seven studies suggested the need for specialty or operation-specific calculators [16,18,19,20]. Interestingly, these studies all reported a moderate to high predictive capability for the ACSRC. Overall, the ACSRC may have some predictability for postoperative cardiac complications. Further research is needed to understand the necessity of operation-specific calculators and the role each risk assessment variable plays in the finalized risk assessment.

## 2. Discussion

The RCRI and ACS NSQIP calculators aid physicians and patients in deciding whether to proceed with the surgical or medical treatment of malignancies. In previous studies, the RCRI, a relatively computationally simplistic tool, demonstrated difficulty in predicting cardiovascular outcomes in patients undergoing several types of operations, particularly intrathoracic resections, head and neck surgery, and partial nephrectomies. The ACSRC demonstrated a more significant application to surgical oncology cases, likely owing to its inclusion of cancer-specific patient factors and CPT code risk values. 

However, both struggled to accurately predict postoperative cardiovascular outcomes, particularly in thoracic and head and neck surgeries. These cases might benefit from the inclusion of additional risk factor inputs, such as predicted operation time, or more tailored patient factors, such as functional vital capacity, and cancer stage values. This emphasizes the limitations of these nimble, readily applied calculators for cancer patients, with examples like the predictive power of serum creatinine in partial nephrectomies showing the potential benefits of condition-specific calculators. 

Although these risk calculators may not be perfectly generalizable to the cancer population, risk evaluation is still possible using more encompassing methodologies. These guidelines have been implemented by the European Society of Cardiology (ESC) and are outlines in the 2022 ESC Guidelines on Cardio-Oncology [23]. The ESC suggests perioperative risk stratification utilizing a risk assessment tool (HFA-ICOS risk assessment) in addition to evaluation of severe or symptomatic cardiovascular disease and neoadjuvant cardiotoxic cancer therapy prior to oncologic surgery [23]. Utilizing this methodology, the ESC Guidelines combine risk assessment tools with additional oncology-specific criteria and presents promising data for perioperative cardiac oncology risk assessments.

## 3. Conclusions

Until enough data are gathered for both the creation and validation of novel preoperative risk calculators, the application of tools like the RCRI and ACSRC will continue to be the mainstay of preoperative assessments. As it is challenging for a single stratification tool to account for all patients, more research should consider specific risk factor stratification or individualized calculators accounting for tumor or therapy type. Therefore, some advice to a prudent provider is to never rely fully upon the predictions of a single tool, but rather to integrate such quantitative data into the patient’s greater clinical milieu and the physician’s understanding of the potential surgery and cancer treatment plan in order to best guide care strategies. 

## Figures and Tables

**Table 1:** The Revised Cardiac Risk Index.

Risk Factor	Values
Elevated-risk surgery	Intraperitoneal, intrathoracic, or suprainguinal vascular = 1, all others = 0
History of ischemic heart disease	History of myocardial infarction; history of positive exercise test; current chest pain due to myocardial ischemia; use of nitrate therapy or electrocardiogram with pathological Q waves = 1
History of congestive heart failure	Pulmonary edema, bilateral rales or S3 gallop; paroxysmal nocturnal dyspnea; chest X-ray showing pulmonary vascular redistribution = 1
History of cerebrovascular disease	Prior transient ischemic attack or stroke = 1
Preoperative treatment with insulin	1
Preoperative serum creatinine >2 mg/dL	1

**Table 2:** ACS NSQIP Risk Calculator.

Variable	Categories
Age group, years	<65, 65–74, 75–84, ≥85
Sex	Male, female
Functional status	Independent, partially dependent, totally dependent
Emergency case	Yes, no
American Society of Anesthesiologists Class	Healthy patient, mild systemic disease, severe systemic disease, severe systemic disease/constant threat to life, moribund/not expected to survive surgery
Steroid use for chronic condition	Yes, no
Ascites within 30 days preoperatively	Yes, no
System sepsis within 48 h preoperatively	None, SIRS, sepsis, septic shock
Ventilator dependence	Yes, no
Disseminated cancer	Yes, no
Diabetes	No, oral, insulin
Hypertension requiring medication	Yes, no
Previous cardiac event	Yes, no
Congestive heart failure in 30 days preoperatively	Yes, no
Dyspnea	No, with moderate exercise, at rest
Current smoker within 1 year	Yes, no
History of chronic obstructive pulmonary disease	Yes, no
Dialysis	Yes, no
Acute renal failure	Yes, no
Body Mass Index Class	Underweight, normal, overweight, obese 1, obese 2, obese 3
Current procedural terminology-specific linear risk	2805 values

**Table 3:** Selected studies comparing the ACS NSQIP calculator for oncologic primary resections.

ACS NSQIP Surgical Risk Calculator for Primary Resection						
Category	Author	Surgery	Number of Patients	Outcomes		Recommendations
**Gastrointestinal**	Alzahrai et al, 2020 [16]	Laparoscopic Gastrectomy	207	**Brier score**		Low predictability for postoperative adverse events. Suggest addition of disease or operative specific variables
				Pneumonia	0.009	
				Any Complication	0.154	
				**C-Statistic**		
				Pneumonia	0.65	
				Any Complication	0.57	
	Vos et al, 2020 [17]	Total Gastrectomy	452	Higher complication rate than predicted ACSRC (45% vs. 29%)		Predictive for cardiac complication, renal failure, death, and discharge to nursing or rehabilitation facility
				**Brier score**		
				Cardiac Complication	0.019	
				Death	0.017	
				**C-Statistic**		
				Cardiac Complications	0.83	
**Head and Neck**	Vosler et al, 2018 [18]	Total thyroidectomy, total laryngectomy, hemiglossectomy, partial glossectomy, laryngopharyngectomy	107	Underpredict Any Complication (30%) and Cardiac Complications (100%)		Potential utility for head and neck oncology. Specialty-specific calculator would adjust for specifics
				**Brier Score**		
				Cardiac Complications	0.028	
**Thoracic**	Chudgar et al, 2020 [19]	Pulmonary Resection	2514	**C-Statistic**		Comparable risk assessment between ACSRC and SURPAS. Continued need for calibration with thoracic surgery
				Any complication	0.728	
				Cardiac Complications	0.821	
				Death	0.753	
**Gynecologic**	Rivard et al, 2016 [20]	Gynecologic Laparotomy	1094	**Brier Score**		ACSRC accurately predicts which patients might benefit from NAT in lieu of surgery due to cardiac complications or death. It does not accurately predict other complications including SSI, UTI and pneumonia
				Cardiac Complications	0.011	
				Death	0.008	
				**C-Statistic**		
				Cardiac Complications	0.708	A tailored prediction model may be needed for gynecologic oncology
				Death	0.851	
	Szender et al, 2015 [21]	Gynecologic Surgery	628	**Brier Score**		Cardiac complications cannot be verified by the ACSRC and risks should be interpreted with reservation
				Any complication	0.023	
				Death	0.004	
				Venous Thromboembolic Event	0.003	
**Musculoskeletal**	Labott et al, 2021 [22]	Proximal Femur Replacement	103	Overall postoperative complication rate (52%) was more than doubled for all CPT codes		ACSRC does not adequately predict complication incidence and should not be used in preoperative decision making
				**C-Statistic**		
				Any Complication for all CPT Codes	<0.576	

## Abbreviations

ACS = American College of Surgeons; ACSRC = ACS risk calculator; AUC = area under the curve; CPT = Current Procedural Terminology; MACE = major adverse cardiac event; MI = myocardial infarction; NSQIP = National Surgical Quality Improvement Project; POD = postoperative day; RCRI = Revised Cardiac Risk Index; ThRCRI = Thoracic Revised Cardiac Risk Index.
